# Modifying clonal selection theory with a probabilistic cell

**DOI:** 10.1111/imr.12695

**Published:** 2018-08-11

**Authors:** Philip D. Hodgkin

**Affiliations:** ^1^ Immunology Division The Walter & Eliza Hall Institute of Medical Research Parkville Vic. Australia; ^2^ Department of Medical Biology The University of Melbourne Parkville Vic. Australia

**Keywords:** clonal selection theory, immune regulation, lymphocyte activation, mathematical modeling, self‐non‐self, two‐signal theory

## Abstract

Problem‐solving strategies in immunology currently utilize a series of ad hoc, qualitative variations on a foundation of Burnet's formulation of clonal selection theory. These modifications, including versions of two‐signal theory, describe how signals regulate lymphocytes to make important decisions governing self‐tolerance and changes to their effector and memory states. These theories are useful but are proving inadequate to explain the observable genesis and control of heterogeneity in cell types, the nonlinear passage of cell fate trajectories and how the input from multiple environmental signals can be integrated at different times and strengths. Here, I argue for a paradigm change to place immune theory on a firmer philosophical and quantitative foundation to resolve these difficulties. This change rejects the notion of identical cell subsets and substitutes the concept of a cell as comprised of autonomous functional mechanical components subject to stochastic variations in construction and operation. The theory aims to explain immunity in terms of cell population dynamics, dictated by the operation of cell machinery, such as randomizing elements, division counters, and fate timers. The effect of communicating signals alone and in combination within this system is determined with a cellular calculus. A series of models developed with these principles can resolve logical cell fate and signaling paradoxes and offer a reinterpretation for how self‐non‐self discrimination and immune response class are controlled.

## 1 INTRODUCTION

Immunology was catapulted forward just over 60 years ago by a powerful idea, the Clonal Selection Theory (CST), articulated and championed by Macfarlane Burnet.^1^ Few today question the impact or genius of Burnet's invention, or that it continues to provide principles and foundations for much of modern immunology. However, this solid platform quickly ran into hurdles that, it is fair to say, have led to a rather inelegant and, from a theoretician's perspective, unsatisfactory collection of ad hoc rules and qualitative heuristic solutions.^2^, ^3^

My personal start in immunology was inspired by Burnet's demonstration of the value of theory and I have been searching ever since for ways to resolve the difficulties CST encountered interpreting modern experiments. However, immunology presents unique challenges to the aspiring theoretician, including an enormous number of working components and copious information gathering at multiple levels, from molecular to whole animal. Inventing ways to compose and organize a modern theory has been a major challenge. However, working with remarkable colleagues and passionate members of my laboratory, I believe that we have found an avenue that seems very encouraging. While a complete theory might not yet be ready, the elements for useful modifications to CST seem to be falling into place. Here, I review the steps taken to formulate this new perspective, combining a bit of history and philosophy with results to illustrate how, even though incomplete, the modifications can solve existing problems of CST. For convenience, I will use the working title quantitative clonal selection theory (qCST) to describe the developing theory.

## 2 HITS AND MISSES FOR CST

What was brilliant and prescient about CST? At the forefront is the depiction of immunity as emerging from the combined action of a population of cells following simple rules. This notion was coupled to the idea that an enormous range of different specificities for receptors could be created by taming stochastic processes within individual cells. The theoretical structure was spare, elegant, and axiomatic in its presentation. CST provided novel and powerful explanations for the long‐standing puzzles of antibody specificity and immune memory, while also addressing the perplexing issue of self‐tolerance by deleting self‐reactive clones. In essence, Burnet identified that immunity required a diverse population of cells and it is their combined cellular intelligence that is required for an optimal outcome.^4^

Hence, what remains unexplained by CST? Two serious challenges to the integrity of CST can be exemplified by twin discoveries by Jacques Miller soon after CST was formulated. The first was a separate class of immunity mediated by thymus‐educated T cells^5^ (reviewed in 6). While this in isolation is not a problem (CST can equally be applied to T as well as B cells), the discovery presaged the unveiling of more and more subtypes of immune cell where each has variations in their activation requirements, effector action, and subsequent response dynamics. This journey of cell type discovery is ongoing and shows little sign of slowing, complicating the application and prediction of cell fates and control with an accumulation of knowledge on each cell. I will refer to this difficulty as the cell type dilemma.

The second challenge was the finding that T and B cells cooperate to generate antibody.^7^, ^8^ We take this so much for granted it is difficult to see why it is so challenging to theoreticians. Why do cells cooperate to make antibody? What is the logical nuance that makes sense for the evolution of such a system? An early attempt to answer these questions, the influential two‐signal theory of Bretscher and Cohn, focused on the question of self‐tolerance and posited the need for cooperation when deciding whether to tolerate a cell or become activated.^9^, ^10^ Two randomly generated receptor‐bearing cells were highly unlikely to both detect self‐antigens at the same time. Thus, one signal (one recognition event) would lead to tolerance, whereas two signals would activate the cell and mount a full response. This clever probability argument resonated with immunologists and was widely adopted.

This requirement for cell cooperation was soon extended to the activation of T cells themselves, although in a different arrangement. For T‐cell activation, an accessory cell delivering an antigen nonspecific “second” signal was required.^11^, ^12^, ^13^ As delivery of these second signals was not tethered to a second antigen receptor, the strict logic of Bretscher and Cohn was bypassed. Nevertheless, a hybrid two‐signal model for T cells was widely adopted.^3^ Cooperation with an antigen‐presenting cell (APC) for provision of second signals is still seen today as controlling the decision between tolerance and activation, although the identity of the two signals and the simultaneous action of, for example, growth factors such as IL‐2 remain imprecise. While textbooks indicate that dendritic cells provide obligatory signals such as CD28, there are many other costimulatory molecules that can substitute, raising the question of how they contribute to a threshold decision around tolerance or not. This difficulty processing large numbers of signals in a rational manner, for each of the many cell subtypes, I will refer to as the signaling dilemma.

## 3 MAKING WAY FOR TOLERANCE, DANGER, INNATE IMMUNITY, AND CLASS

There are theory‐based explanations for why lymphocyte activation has evolved such complex requirements. One, presented initially by Charles Janeway,^14^, ^15^ proposed that the decision to activate or not must pass the requirement that the threat must stimulate the APC by first engaging an innate system of germline‐encoded pathogen‐associated molecular receptors. This theoretical argument was rapidly supported by the discovery of large numbers of pathogen‐specific stimuli that can activate dendritic cells and enhance the generation of second signals.^16^, ^17^, ^18^, ^19^ Another related theory, developed by Polly Matzinger, argues that the APC responds to “danger” signals such as the detection of dying cells.^2^, ^20^ Both views take the position that the immune system need not respond to everything that is foreign but can focus on those insults that appear to be posing a serious threat. Both make important predictions and have garnered strong evidence in support.^2^, ^16^, ^17^, ^18^, ^19^, ^20^

An alternate explanation for signal complexity, not tolerance related, is that the many intercellular signals are needed to control the choice of immune assault directed to a pathogen. Typically, a given immune response motivates only a subset of the many possible immune effector types available. These decisions regarding immune response class are well known to be influenced by regulatory intercellular factors (such as T‐cell cytokines or accessory cell‐derived costimulatory molecules) that alter the rate of differentiation outcomes.^21^, ^22^, ^23^, ^24^

The cell type and signaling dilemmas, already apparent two decades after CST, have become more extreme with advances in technology. Modern tissue visualization and single‐cell measurements at molecular resolution dramatically increase our awareness of the uniqueness of every cell and the complexity of the system in situ. T and B cells express multiple receptors and receive modifying signals from many other cells, including lymphocytes, dendritic cells, macrophages, and local stroma. These signals can modify activation, proliferation, survival, migration, differentiation, and ongoing effector responses. There is so much complex structure in, for example, a lymph node, or the bone marrow, that the prospect of understanding and modeling all of the cellular and molecular details governing each cell fate can seem insurmountable.

## 4 BEGIN AT THE TOP: LOOK AT THE PARADIGM

Numerous examples from the history of science teach us that successful theory construction depends on choosing, and sometimes inventing, an appropriate framework—a paradigm—from which to work.^25^ A paradigm serves effectively as a logical template for how a formal theory for the system could be constructed. According to Kuhn,^25^ scientific investigations are always conceived within such a framework, even if unacknowledged. It is fair to say that there are many opinions within our immunological community as to the appropriate paradigm for immune investigations. However, two alternatives dominate the discourse. The first I will call the “subset” paradigm that aims to identify and subdivide cells into categories and define rules for their behavior under different conditions. Within this framework, prediction requires Boolean style logic to deduce consequences from this accumulated knowledge. This approach, exemplified by the various versions of two‐signal theory, captures and codifies accumulated wisdom but is difficult to apply systematically and consistently and cannot easily include time necessary for translation to dynamic models.

A second paradigm, I will call it “strong determinism,” is consistent with some modern “systems biology” approaches, and aims to build high‐dimensional deterministic models from advanced, comprehensive knowledge of all ongoing molecular and cellular processes. The presumption is that if we can know the starting points, and how all the molecular pieces interact, we might be able to make predictions with mathematical precision. Even rudimentary knowledge of modeling suggests that this approach is going to be difficult and immensely overparameterized. Worryingly, this approach also has clear parallels with unsuccessful deterministic programs from physics and will encounter significant difficulties if the immune system relies on stochastic events.^26^

In summary, to build an immune theory suitable for quantitative modeling, we must choose an appropriate paradigm, and for some time, our adopted candidates have been running into logical difficulties that explode in complexity with further investigation. We find ourselves at a crossroad. The problems could simply reflect a lack of knowledge and a satisfying solution will become evident after further experimental dissection. Alternatively, it is possible that our current paradigms are inherently unsuitable and headed to a dead end.^26^ It seems that there is no simple way to assess which situation we are in; the system is so complex, any experiment can be interpreted in different ways. However, given the continuing problems, it seemed prudent to search for and explore alternatives.

## 5 EVIDENCE FOR RANDOMIZING PROCESSES AND CELLULAR COMBINATORICS

Hints that both the subset and strong deterministic paradigms might struggle with inherent stochastic processes within hematopoietic cells and lymphocytes have been glimpsed for some time without gaining a lot of traction in the field.^27^, ^28^, ^29^, ^30^, ^31^, ^32^, ^33^ For our program, a major clue for where and how stochastic processes might be operating came after the development of division tracking methods.^34^ Careful experiments observing cells changing fate suggested that random combinations for alternative fates had been followed in each cell. While individual B and T cells had highly variable division times, the proportion of cells that altered their effector functions was fixed for any generation at any time.^35^, ^36^ Thus, the cell machinery responsible for variation in time to divide seemed to be operating independently of the cellular machinery controlling isotype switching, or cytokine release, after each cell division. Additional division‐linked fate changes, such as a second switch event, or development into plasmablast cells also proceeded independently in the same cells ensuring simple combinations could predict the proportion of multiple cell types emerging following stimulation in both mouse and human cells.^37^, ^38^, ^39^, ^40^, ^41^, ^42^ Cytokine signals that affected fate often altered the probabilities of events already underway to manipulate the proportion of cells of different types.^37^, ^38^, ^42^ Furthermore, experiments found that cell machinery governing variation in times to die is also regulated independently of division.^43^ Together, these results provided the basis for a statistical paradigm for cell construction based on a proposed “law of independence.”^43^ Cells operating and constructed according to this law use multiple independent cellular “machines” to assign fate changes and easily generate a broad range of heterogeneous outcomes. Furthermore, tuning and regulating the generative stochastic processes themselves would manipulate the mix of cell types created without needing control over every cell fate.^42^, ^43^

## 6 NONLINEAR RESPONSES AND CAUSATION

The proposal that heterogeneous cell types might be generated as part of an autonomous cellular program, stood in contradiction with a strict interpretation of the subset paradigm that presumes each unique cell type results from identifiable, external signaling guidance for its formation. For example, Th2 T cells require IL‐4,^44^ or IgG2a‐secreting B cells are generated by interferon‐γ.^45^ A new paradigm must reconcile autonomous generation of multiple cell fates with the substantive evidence for the role of signals in cell differentiation. Review of the experimental evidence suggested a way forward.^36^, ^43^, ^46^ Typically, regulatory influences have multiple effects on cells (altering proliferation, cell survival as well as differentiation) and such compound effects are extremely difficult to deconvolve into component parts. Furthermore, common assays used to measure effects of cytokines and gene changes are usually highly dependent on cell proliferation, whether in vitro or in vivo, enabling small effects on, for example, division rate and/or survival to be read out as a large difference in final cell number. As a result, experiments showing a large “causative” outcome might be incorrectly interpreted. The alternative interpretation, that an ongoing process is markedly amplified (or diminished) by the new signal (or molecular change), requires interpretation with an appropriate parametric model that captures and recreates in silico, the internal cellular machinery driving the final outcome. Therefore, until we have such a quantitative framework, we should be cautious and hold in check definitive conclusions around the precise role of components (transcription factors, cytokines, costimulatory receptor ligands) classed as obligatory, or causative of differentiation, or activation outcomes.^42^, ^43^

## 7 HIERARCHIES OF BIOLOGICAL MACHINES

Historical examples demonstrate that an important task when building a theory is finding the requisite explanatory level. Immunology challenges us, however, with comprehensive information at multiple levels. Our experimental campaigns are operating at molecular resolution and any putative theory that uses nonidentifiable conceptual constructs, such as “division timers” or “division counters,” will appear imprecise and inadequate compared to a molecular‐based solution. Thus, to defend a putative paradigm for CST operating at cell level, we need effective answers to questions such as: (a) How do we incorporate information into a theory at multiple levels simultaneously? (b) Will an eventual theory emanate from the molecular level and supersede cell‐based theories? (c) How can a predictive theory of the cell give us the ability to predict the effects of drug therapies or gene changes and other molecular interventions?

Immunologists, myself included, are not usually comfortable straying into epistemology, but this is where we must head. The answer I developed for our research program drew on the observation that we always “understand” a system with a mechanical description operating one organization level down.^47^ For example, the theoretical basis for chemistry is the explanation of bonding properties of atoms. A theory of bonding properties of atoms is explained by the properties of subatomic particles. Thermodynamics is explained by the properties of populations of particles not individual particles themselves. Theories that attempt to translate knowledge across multiple levels become overly complex because information from two levels down can always be summarized more efficiently into rules for the mechanical operators found one level down.

Since Burnet, immunity has been identified as falling into the domain of cell population dynamics. Hence, by the “one level down” rule, a theory based on the cell depicted as a logical machine is an appropriate explanatory level. Following this same line of argument, to explain the molecular machinery of the cell, we need a separate theory. This theory would give the molecular detail for how the logical components operated, such as the timer, the counter, or the attendant randomizing processes. By completing theories at both levels, the effect of molecular changes (such as gene mutations or drug exposure) could be translated from the lower molecular level to explain specific effects on cell operation. For example, a drug might alter division time by 20%, and this information can be directly incorporated into the cell level theory to predict effects on population‐based immunity. To avoid confusion regarding the levels of theory, I will call a theory of cells that predicts cell population dynamics as a theory^C^ and a theory directed to molecular descriptions of cell behavior as a theory^M^. I envisage that the two theories can be designed to efficiently translate results from one to other. This use of two theories to solve the dilemma posed by multiple levels of information is mirrored in the original structure of CST.^47^

## 8 CONJECTURES AND BURDEN OF PROOF

The discussion so far has broadly outlined details for a paradigm focused on the independent operation of modular machines assorted randomly within cells. When these ideas were first proposed,^42^, ^43^ they simply supported a conjecture that they might be sufficient to construct a new quantitative theory. As a proposer, the burden of proof fell on my shoulders to flesh out details and confirm that the ideas could resolve logical dilemmas as speculated. While the paradigm looked promising the enormous number of ways it could be realized by cells was daunting and could not be solved intuitively. As a result, testing this conjecture quickly became a major activity in my laboratory. We focused on reductionist style investigations to examine and refine the underlying behavior of cellular fate control and learned to use this information to construct deductive models to test consistency with immune features, in vitro, and where possible, in vivo. While there are still many experimental details required to complete this theory, the results so far are encouraging, and the following principles are emerging with strong support as suitable foundations for qCST.

## 9 EMERGING PRINCIPLES OF CELLULAR MECHANICS

### 9.1 Intracellular machines and independence

The critical primary axiom is that every cell can be defined as comprised of a mix of “machines” governing different operations, such as time to divide, time to die, or initiating a fate decision.^42^, ^43^, ^48^, ^49^ Furthermore, many of these machines are capable of autonomous and independent construction, operation, and regulation within the cell. For this reason, alternative fates, such as division or death, are often found “in competition” within the one cell. Complete independence is not essential for this theory, however, to date, evidence from direct filming, and the accuracy of models has proved consistent with this assumption.^49^, ^50^, ^51^, ^52^ Any version of qCST must formally define and identify the types and operation of such cellular machines as well as the rules for their regulation and changes with time, signaling inputs, and impact on the other machines within the cell. I shall refer to this formulation as the system's cellular mechanics.

### 9.2 The probabilistic cell

The second‐defining axiom is that when created, all cells, and all functional mechanical modules, are manufactured by cellular processes that can be manipulated by stochastic modifiers that affect the performance within the cell (manufacturing randomness). Similarly, the operation of each cell machine could incorporate stochastic steps that diversify otherwise identical cells (operational randomness). It is a key assumption that these stochastic processes have been introduced and tuned by the evolution of the system and are inherent and necessary for successful operation of the immune response. Thus, the net result is to diversify the fates of stimulated cells even under identical conditions and ensure that successful immunity requires a collection of activated cells, even if they belong to the same cell type.^42^, ^43^, ^49^, ^50^, ^51^, ^52^, ^53^, ^54^

### 9.3 Combinatorial uniqueness

Experiments that measure times to fates in similar cells, or timed response differences, usually conform to probability distributions that are right‐skewed such as the lognormal distribution.^41^, ^43^, ^48^, ^49^, ^50^, ^51^, ^55^, ^56^, ^57^, ^58^ The randomness of each element, and the independence of operation in each cell, means, effectively, a population of cells once generated has not only predictable features but also that any individual cell is unique. Thus, cells are capable of extraordinary heterogeneity, and this can be captured and recreated by using appropriate probabilistic models.^43^, ^49^, ^51^, ^52^

### 9.4 Cellular calculation

To allow regulation of immune responses, the internal functional machines are each modifiable by both external signals and by intracellular reprogramming after some fate change. This fate change may turn on, off, or change the time to fate of other function controlling machines in the same cell. Operations of internal components can alter other components. For example, division can alter the likelihood of isotype switching 35, 37 or reset the time to die.^49^, ^51^ In other situations, recently discovered, division will have no effect on the time to stop dividing or the time to die.^59^ This latter rule should lead to families that share timed events (such as division cessation or death) in the same generation,^59^ as is frequently observed by experiment.^50^, ^60^, ^61^ Given the mechanics and rules of interaction between components, and the effects to be altered over time, a calculation scheme to model the population cell dynamics in number and type is possible. As processes are operating and dictating cell changes over time and require signal *integration* and affect *differentiation*, it is reminiscent of the calculus, and with Amanda Gett, we labeled these operations as the *Cellular Calculus*.^43^

### 9.5 A repeated canonical program

T and B lymphocytes are different cells with nonoverlapping functions and modes of regulation. However, evidence, to date, suggests that the same cell mechanical principles can be adapted for both cell types. While receptor inputs are changed, the internal modular processes governing fates appear similar. Typically for each cell, many investigators have found the features of the adaptive response including proliferation, differentiation, division cessation, and cell death, unravel as an autonomous cellular program after the initial stimulus.^59^, ^62^, ^63^, ^64^, ^65^, ^66^, ^67^, ^68^, ^69^, ^70^ These patterns and similarities offer an insight to the genesis of the adaptive immune system, as arising from a single primitive program, perhaps related to cellular machinery governing embryogenesis or organogenesis.^64^ If correct, there would be two directions for the evolution of alternative immune strategies from the one primitive program. The first is to replicate and create new specialized lineages of adaptive response (such as T and B) requiring a change in the initiating receptors and the fates that are connected downstream. Second, the fates allocated as the response unfolds (for example, a change in isotype) can be modified and reconnected to new outcomes. As a consequence, different cellular responses and activation regimes are related by common mechanical control units and can be captured as variants by the same theory. Presumably during evolution, patterns for allocating cells to different classes after activation and receiving inputs to regulate the strength and type of response have been created, tested, selected, and optimized for best effect. Such canonical programming, independent of the receptors being used, is consistent with finding similar cell lineages with completely different receptor systems in primitive species.^71^

## 10 QUANTITATIVE MODEL EVOLUTION AND COMPLETENESS CONJECTURE

If the above principles correctly capture the underlying cellular operations, then an appropriate arrangement can be framed into deductive models to predict dynamic changes in cell numbers and types over time. However, correctly framing the effect of different conditions such as cytokine exposure cannot be intuited and must be determined by experiment. Improving models iteratively will require working from simple scenarios such as highly controlled in vitro responses of T and B cells, to predicting more and more complex arrangements including, ultimately, in vivo predictions. That is, to move effectively from the first‐order responses in vitro (no cell interaction) to higher order interacting scenarios is a path to completing a deductive, quantitative version of qCST. The theory is well suited to techniques of model development, including mathematical equations, numerical solutions, and agent‐based models and all may have their suitable place in different situations.

## 11 TESTING qCST MODELS

While still evolving with experimental advances, a series of models developed since 2000 offer insight for how logical difficulties with two‐signal theories can be resolved with qCST.^43^, ^48^, ^49^, ^51^, ^59^, ^63^, ^72^

The first model, developed with Amanda Gett, recreated asynchronous division peaks for T‐cell proliferation and could be fitted to CFSE time series to extract average division times.^43^ Analysis of multiple stimulatory conditions confirmed that cytokine and costimulatory signals could add together to modify times to first and subsequent division times. We found three relatively small reductions in time to divide by anti‐CD28 and IL‐4, added independently to predict a net outcome of 10‐fold more cells after 4 days of culture, a figure matched by experiment. Thus, we concluded that costimulatory signals were not obligatory, but could be made to appear so with single time point, qualitative experiments. Thus, with this first attempt, we were able to challenge the basis of two‐signal theory and call into question binary views of tolerance. In a further analysis with Elissa Deenick, the cytokine IL‐2 was found to create a highly nonlinear relation between concentration produced after stimulation and the resulting effect on proliferation.^48^ These experiments highlighted the difficulty of interpreting T‐cell proliferation experiments when IL‐2 is uncontrolled in culture, as is often the case.

In 2007, with Edwin Hawkins, Marian Turner, Carel van Gend, and Mark Dowling, we introduced a model that hypothesized a resetting and redrawing of randomized division and death times upon each cell division.^49^ This model added a third independent cell mechanical component, a division counter controlling division progression, until reaching a cell's “division destiny,” suggested by experimental observations.^73^ We called the combination of mechanical components controlling division and death, the cell's “Cyton.” The model was described with differential equations and solved with numerical algorithms to illustrate the many available response patterns that were theoretically possible with changing stimulation strength. Vijay Subramanian and Ken Duffy later produced a version as a branching process that allowed higher moments to be calculated.^74^ The Cyton model had the satisfying feature that the typical pattern of immune responses, where cells proliferate, stop dividing and die, often over a long period, could be naturally created from cellular machinery without any further directed guidance along the way. The Cyton model also illustrated how signal integration affecting division times, and times to die, could sensitively alter the response from weak stimulation, leading to the dominance of clonal deletion, to strong responses, where cell numbers become very large. While the two extremes could be viewed as binary outcomes, equivalent to tolerance or activation, any intermediate level was also possible.^49^

The evidence for division counting before returning to quiescence, built into the Cyton model, was drawn from B‐cell experiments.^50^, ^73^ With Julia Marchingo and Susanne Heinzel, we asked if T cells might also count divisions if the autocrine growth factor IL‐2 was inhibited. Under these conditions, stimulation‐dependent control over division progression was revealed and many costimuli, and cytokines were found to affect the number of divisions T cells completed before returning to quiescence.^63^ Quantitative experiments determined that combinations of signals added linearly for division number, resulted in predictable geometric effects on total cell yield. These results again confirmed the view that costimulatory signals are not obligatory for T‐cell activation but can appear so by experimental design. Furthermore, as costimulatory and cytokine signals affecting T‐cell responses can be derived from many sources such as dendritic cells, macrophages, other T cells, NK cells, or other innate detection systems, the results argue against the view that T‐cell activation requires carefully prescribed signaling requirements. Rather, we argued that many combinations from multiple sources can achieve a similar strong responsive outcome.^63^

My final example incorporates a modification that arises from the discovery by Susanne Heinzel, Andrew Giang, and Lynn Corcoran that the controlled burst of division progression by T and B cells after activation is governed by the rise and fall of Myc.^59^ Curiously, the pattern of Myc loss over time was unaffected by cell mitosis and was carried through into successive generations. This result identified ongoing division progression as properly controlled by a heritable “timer” rather than the division counter employed in the original Cyton model. The two modes of regulation yield similar outcomes if time of division, after the first, is relatively uniform, as they are for CD8 T cells.^58^ A heritable timer mechanism for passing fate change information through successive generations was also noted for the time to cell death.^59^ A modified version of Cyton cellular mechanics that incorporates timed, heritable settings for division destiny, and death on activation, is particularly well suited to modeling stimulation conditions that evoke the progressive autonomous responses of T and B cells. This revised model is again highly sensitive to small parametric changes and, as for the original Cyton model, progressive increases in input number and strength can titrate responses from rapid net loss of activated cells to rapid net increases.^59^

To summarize, this series of experiments and cell mechanical models support the conjecture that T‐ and B‐cell responses are regulated quantitatively and that immune responses are hypersensitive to even small modifications by cytokines and costimulatory signals, making experiments difficult to interpret without quantitative tools.

## 12 A QUANTITATIVE INTERPRETATION OF SELF AND FOREIGNNESS WITH qCST

I would like to change perspective now. Instead of arguing the case *for* qCST, I will take the evidence so far as sufficient to conclude that a powerful theory built on these principles is possible. With this new perspective, I return to the two‐signal theories to examine them more closely. In most versions, a T‐ or B‐cell meeting antigen is forced to make a critical decision: die for tolerance or become activated for an immune response. While this decision might require additional inputs, perhaps from the innate immune response, or other sensors of “danger,” these theories are expecting a mode of signal integration that dictates this first decision as one of two choices. As there are many potential signals that affect this decision, mathematical models will require the identification of a signal‐processing calculus to sum the inputs and govern the binary outcome. To date, how such complex cellular calculation operates has not been determined in any satisfactory, accurate manner.

By changing paradigm to qCST, this complex signaling dilemma is solved by removing the expectation for binary decisions completely. This can be illustrated by the Cyton model. In this model, activation signals motivate changes and reprogramming of both the division and death time controlling cellular modules within the same cell. The individual cell does not “choose” or process a signal, or combination of signals, into a single decision—divide or die—both options are in operation and being pursued in the same cell and the final outcome for single cells will vary depending on which fate timer fires first. The important difference from the two‐signal viewpoint is that the cell is *not* forced into a decision. It simply begins responding: the total sum of the inputs will ultimately dictate the net outcome for all cells.

This satisfying elimination of decisions and prescriptive control of fates can be extended to include immune response class. Division tracking experiments have identified a close integration of division progression and choice of response class changes such as antibody isotype and cytokine secretion. Thus, class and response strength, indicated by how many divisions are completed, appear to have evolved to be in step with each other.^35^, ^36^, ^37^, ^38^, ^39^, ^40^, ^42^, ^75^ These two seemingly different processes turn out to be inseparable and enmeshed. Thus, as a further principle for qCST, I suggest that decisions governing tolerance, the strength of response and immune response class, are all part of the same cellular programming and cannot be divided into different parts and separate theories.

To summarize, in qCST, signals from cytokines and costimulatory molecules directing activation decisions of T and B cells should be viewed as units of information. These units always simultaneously transmit information that a threat has been detected and include class information. Thus, different inputs derived from many potential sources (ie, APC, NK, or innate cell‐promoted inflammation) can modify the outcome and no exact combination of signals is required. In short, self/nonself and class are part of the same equation and should not be segregated. Signals that affect one will almost always have an impact on the other. A further useful conceptual viewpoint is that the inputs are being summed in consideration of how foreign or dangerous is a given threat. As a general rule, these inputs are summed linearly, but due to the hypersensitivity of the proliferation dynamics, the outcomes are translated into exponential, and greater, differences in cell number.^43^, ^48^, ^49^, ^59^, ^63^, ^76^

## 13 REVISITING OTHER THEORIES AND EMPIRICAL RULES FOR IMMUNITY

It is important to show that a new theory is consistent with, and can replace, earlier options. The logic of Bretscher and Cohn that two antigen recognition events will increase the likelihood of foreignness can be viewed as partially true in qCST: the more lymphocyte recognition events initiating the response, the greater amplification of the outcome. For this reason, it seems highly likely that antigen‐stimulated B cells that fail to find T help represent a significant path to tolerance. As a result, the two‐signal logical rule can retain its original explanatory appeal.

Similarly, the logic of the Janeway and Matzinger theories for the role of innate recognition and detection of danger and dying cells is also accommodated within the nonlinear consequence of adding stimulatory signals. However, this support comes with the qualification that such inputs are not essential or obligatory but can appear so under some experimental conditions. Nevertheless, qCST allows both perspectives to operate simultaneously, and it seems likely that such signal regimes play important roles for natural immunity and tolerance preservation.

In addition to the theoretical arguments that capture immune decisions within a unifying rule, there are a collection of other empirical “rules” or pathways for immune cell control that comprise our collected knowledge of immunity. It remains to be demonstrated that all such experiments can also be accommodated and reinterpreted within qCST. However, as an example, it seems clear that obligatory T cell help for cytotoxic T cells mediated by IL‐2 production by a T‐helper cell or by indirect activation of an APC^77^, ^78^, ^79^ is easily reconciled by sensitive nonlinear consequences of signal addition.

In some model systems, self‐antigen‐stimulated cells adopt an anergic state rather than dying.^80^, ^81^ Anergic cells are partially activated, but die after a few days for lack of further signals.^82^ These features of anergy fit well with the continuum of activation allowed by qCST, although integration of kinetic details into the model will require further experiments.

## 14 PROGRESS ON THEORY^M^

The strategy of dividing our theoretical goals into development of two nested theories for multiscale immune modeling provided a license to focus on developing versions of cell level mechanical models without needing to identify the molecular machinery underlying such components. However, completing qCST will also require the development of the lower level theory^M^. While this level theory is far from complete, it is useful to review some interesting features at this point.

### 14.1 The source of randomness

The cellular machinery in qCST generates heterogeneity in two ways. There is the variation in component performance (ie, variations in times to fate in each cell) and there is the combinatorial possibilities of distributing these varying components randomly among individual cells and playing out the consequences upon activation and proliferation. How is this cellular randomness achieved? One likely answer is familiar to all immunologists who use flow cytometers. Individual cell variation in any measured cell component is typically broadly distributed, often lognormally, covering a 10‐ to 100‐fold expression range. These differences apply to any cell feature including surface receptors, signaling molecules, and internal components such as transcription factors. When trying to allocate cells into groups using the subset paradigm, these differences are inconvenient and frequently ignored or downplayed. However, the quantitative version of CST is looking for and expects sources of individual differences, and therefore anticipates these variations and assigns them a great deal of importance. Effectively, it means that construction of otherwise similar cells contains quantitative differences in receptors and components that will alter their subtle perception and fate under identical stimulation conditions.

Evidence that these molecular “construction” differences are significant is highlighted by the finding of remarkable fate similarities between siblings and families in vitro and even in vivo.^50^, ^51^, ^52^, ^60^, ^61^, ^83^, ^84^ It appears if cells are molecular “clones” they behave almost identically, and that the large amount of diversity between similar cells might presumably be traced to a set of molecular expression differences. As a consequence, the fates might be predictable with quantitative measurements of molecular components. As epigenetic mechanisms likely dictate this diversity, this discussion identifies such processes as major contributors to the operation and evolution of the system.

In addition to these examples of cell‐manufacturing differences, cells are also likely to have used and tuned other mechanisms for introducing randomness to diversify similar cells. Transcriptional noise or cellular allocation on division or bifurcating chaotic switching for networks may also be used and exploited in biological systems.^85^, ^86^, ^87^ An example from immunology is heritable stochastic expression of cytokines by a cloned T‐helper cell.^88^ A compelling feature of any complex probabilistic system is that all the sources of variation feed into and can be added to the final description of the functional component they affect. This is a particularly powerful result for translating models from the molecular to cellular levels. The myriad sources of variation contributing to the final expression level of say a receptor, will, in our cell theory, be summarized into a new distribution for the variance within a particular cellular function under similar conditions, this will be illustrated below with a toy model.

### 14.2 Timers and counters

Many mechanical operations in cells can be described as fate timers, such as those seen to determine time to divide and time to die. While often the exact processes are unclear, there is evidence that expression level of critical proteins has an influence over these times, and that therefore, regulation of molecular levels will be a way of manipulating timed outcomes. For example, the time to die of B lymphocytes conforms to a lognormal distribution that can be altered by Bim or Bcl‐2 levels.^49^ The time allowed for division progression is governed by the level and loss of Myc protein.^59^ Thus, as a general rule, manipulation of such levels by signals and variation by epigenetic modifiers might go a long way to explaining the regulation of the cellular mechanics exploited in theory^C^.

## 15 A TOY EXAMPLE OF CELLULAR MECHANICS AND CELLULAR CALCULUS

**Figure 1:**
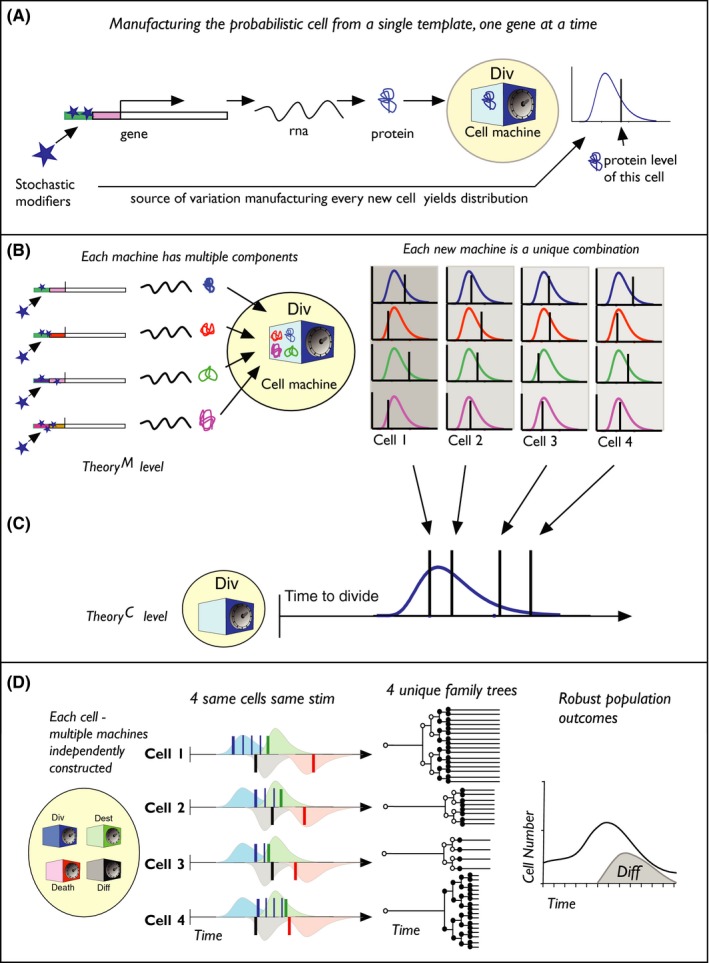
Manufacturing a probabilistic cell at two levels. A, Gene modification for a protein utilized for cell division. A stochastic driver adds, or removes, a series of epigenetic marks that will affect the rate of translation of the gene under all future conditions and hence the level of protein contained in the cell machine *Div*. The level is indicated by the bar on the plot overlayed against the distribution found for a large number of *Div* constructions. B, Illustrates *Div* is comprised of a number of independently randomized proteins as shown at right with bars and distributions for four created *Div*. C, The net effect of the determined level of all the constituents of *Div* leads to variation in the time to divide when called into action. Bars indicate the division time of cells with Div constructs 1‐4, overlayed with distribution from a large number of *Div*'s. D, The final cell is comprised of multiple machines each subject to similar stochastic genesis. The resulting fates for four cells from the same manufacture are shown. Blue bars show division, green shows destiny time, black bar is differentiation, and red is death. These allocated times yield disparate family lineages. Irrespective, cell responses from a number of similar cells yield predictable average outcomes and reliable generation of a mix of cell types

It may be helpful at this point to illustrate the consequences and operation of cell mechanics with a simple, toy example (Figure 1). In Figure 1A, the potential for every mitotic event (a cell “manufacture”), to introduce a “tuned” (ie, by evolutionary selection) level of stochastic influence, is illustrated for the product of a single protein that will play a role in the division time controlling machinery when called into action (the *Div* machine). All cells from the same “forge” are produced by an identical process; however, the exact result for any individual, while determined (shown by the bar on graph), will be different for each cell and the collection of outputs will form a distribution that can be viewed as summarizing the output of the original stochastic process. Here, the process is implicated as an epigenetic marking of the promoter, but many other influences could suffice here. Figure 1B illustrates that *Div* contains many such protein components, all subject to one or more related or independent, stochastic inputs that affect the level in *Div*. The net result is that every time a new cell is made from the same forge, cellular construction of *Div* will vary and hence, the performance of *Div* when activated under identical conditions will yield a determined outcome (the bar) for a single cell, and the population will be described by a distribution. Importantly, as noted also above, the population distribution has summarized all of the lower molecular level stochastic drivers, into a single, easily parameterized result that can now be used for model building to translate from cell to population.

In Figure 1D, we now add in additional cellular machines, the Death timer (*Death*) and a Division destiny timer (*Dest*) to complete the three Cyton components. Each of these machines has been constructed in a similar manner with input from multiple stochastic drivers. The fourth machine, a putative *Diff* timer, will direct the cells to differentiate at a particular time. In this example, the timed effects of *Death*,* Dest*, and *Diff* are considered heritable and transmitted faithfully even if cells divide. In our cell operations, *Div* varies times to first division and then motivates a series of uniform subsequent divisions until the destiny given by “*Dest*” is reached. The fate change is triggered by *Diff* at the given time (this is illustrative and could be expression of a new receptor, or development into a new cell type, such as a plasmablast or a memory cell). Any number of additional modules for simultaneous fate regulation could be included in related arrangements of cell machinery.

The predicted response of four similar cells from the same manufacturing process is illustrated in Figure 1D. Some activation event has turned on or reprogrammed each cellular machine and set each to trigger at particular times. Due to the constructional differences, the choice of time, for each fate, is different and drawn independently of the choice for the other machines. The determined outcome for the individual cell is contrasted to the expected colored distributions for a population of similar cells. As a result of the random differences in each machine operation, and the combinations, each cell will respond differently and trace out distinct clones and fate changes over time as depicted. Despite the highly disparate nature of single‐cell lineages, the population average for proliferation and generation of new cell types will become more and more predictable as the number of cells drawn into the response increases.^51^, ^52^, ^54^, ^74^ Such population averaging from stochastic cellular drivers governing single‐cell variation was first suggested for hematopoietic stem cells after classic studies and theoretical insights from Till, McCulloch, and Siminovitch in 1964.^27^ The remarkable lineage trees depicted in Figure 1 are not dissimilar to those seen in real data (ie, tree diagrams from 50, 89). It is important to look back to the creation of these cells and note that the generative stochastic processes for protein level expression percolated up and collected into the final probabilistic outcome, like streams and rivulets collecting into a final, mighty river. It becomes clear how those lower level stochastic processes could be manipulated by a level of control and by evolutionary selection to have significant effects on the performance, response, and rate of allocation of cells to new types following stimulation.

A second highlight, from reviewing this system, is to note how by taking advantage of stochastic processes coupled with intracellular combination, it manages to create enormous diversity without needing to code for, or create, unique conditions for every new cell individually. As a consequence, appropriate deductive models that match the cellular operations can accurately recreate the dynamic evolution of immune responses. Furthermore, the probabilities describing the cell machine model performance have captured completely all the molecular sources of variation that might include translation rate, degradation rate, or limiting enzyme activity without having to identify their source.

## 16 CAN WE ELIMINATE PROBABILITIES?

The principles of qCST attach a great deal of significance to the controlled application of randomizing processes for cell construction and for the operation of cellular functions. As a result, models developed on qCST principles will necessarily incorporate probability distributions to describe and capture the range of outputs from the stochastic events. This reliance on probabilities is often queried with the argument that probabilities are only needed when information is missing. The idea being based on the view that if all initial conditions were known, the result of a coin toss, or roulette spin, could be predicted and hence is not truly random. However, it should be clear that this argument is flawed if the system has evolved to take advantage of stochastic processes and relies on the randomized results. To attempt a deterministic interpretation for such a system is likely impossibly complex and ultimately missing the point for how the system operates.^26^ In drawing this conclusion, note that variations in fates and sensitivities will be, to some extent, traceable to differences in an individual cell created by epigenetic differences in expression levels of molecular components such as receptors or prosurvival proteins. Thus, even though the differences arise as the result of stochastic processes, future individual cell fates may be identifiable if key molecular levels can be measured. This conclusion provides an interesting point of contrast to particles in quantum mechanics that are considered probabilistic and their fates therefore “indeterminate.” I would argue that for theory^C^, individual cells can also be considered indeterminate, in the same way as particles, as our models will not need to predict the fate of every individual cell. However, we can contrast this interpretation against successes of molecular determinism where there is considerable optimism that a comprehensive molecular model of an individual cell could explain and predict its fate (at least until the next, manufacturing, operational, or environmental stochastic event) under given conditions. This deterministic viewpoint is likely to inform and dominate the completion of theory^M^ providing a partial reconciliation between rival probabilistic and deterministic perspectives (although descending further levels will likely revisit the same issues).

## 17 LOGICAL ADVANTAGES OF A PARADIGM SHIFT

When ancient Greek astronomers began taking accurate measurements of planetary motion theoreticians of the day were forced to invent increasingly convoluted models to fit the data within the paradigm that the Earth was at the centre and all motion should be circular. Each improvement in accuracy forced a new model. In a proposal by Ptolemy in the second century AD, 39 orbits and epicycles were required to describe the sun, moon, and five planets. Of course, these additional cycles disappear with a change in framework that places the Sun at the centre and allows orbits other than circles (discussed in 90).

I have argued here that something similar is occurring in immunology with one of the culprits being logical epicycles that arise from strictly applying the subset paradigm to a system that has evolved to take advantage of randomness with the probabilistic cell. The subset paradigm expects cells to fall into discrete categories. Each such category will have defining features and rules of behavior that include their phenotype, their response, and their transition to other states. If qCST is correct, a number of problems can be envisaged with subset paradigm‐based experimental programs. Within qCST cells of a similar subset, such as naïve T cells, exhibit individual differences that ensure diversification arises from the collective outcome of a group of members. For this reason, cells in the same category are capable of seemingly contradictory behaviors. Cells of the same type, with the same stimulation, can both divide and die, switch to different response classes or become an effector and a memory cell. If viewed through a subset framework, this is illogical and will lead to an attempt to identify the source of differences. A further area of difficulty for the subset viewpoint arises after activation and transition to new cell types. The subset view expects to identify clear stages and steps along the way. If different fates emerge from the same cell type, the presumption is that they must have been created by some bifurcation driven by changes in differentiation signals. In contrast, probabilistic cells can and will take multiple paths to the same differentiation endpoint. Identical cells of type A with overlapping probabilities of becoming B, C, or D might be observed to differentiate to cell type C through an intermediate state B or go directly to C or take an entirely different route to cell type D (presuming, for this example, the fates, once achieved, do not feedback and alter probabilities of other fates). Many variable paths are possible and will have, if probabilities are known, predictable frequencies in the response of a population of single cells. Thus, a conventional subset‐based program of research might find itself in a never‐ending attempt to define new cell types and find rules for their generation and regulation. However, this search will be, ultimately, incapable of reaching an endpoint solution for why and how it is so complex. It will also be incapable of developing accurate dynamic predictive models, which I hope we agree, is the ultimate goal for our scientific endeavors. qCST by adopting a new logical paradigm finds a simple way out of the projected subset and signaling dilemmas as differences are expected for every cell and no cell is likely to follow exact average behavior across multiple points of reference. Thus, a switch to a slightly more complex logical framework can lead to a simpler interpretation of our complex immune system.

## 18 PARSING DATA THROUGH A NEW PARADIGM: A CONJECTURE

A barrier to changing paradigms is that the published knowledge and experimental data are designed and interpreted within the one dominant, and now questioned, viewpoint. Thus, there are many articles that report a cytokine or molecular component “causes” differentiation to another cell type, or that a particular helper cell type is essential for a particular immune reaction. This language and such conclusions are called into question by qCST which anticipates small differences to be markedly magnified, or diminished, by the hypersensitive proliferative systems. Of course, causation is possible but will be difficult to distinguish from amplifying or dampening effects. By this argument, much of our knowledge for the effect of molecular and cell components in immunity may have to be reassessed and parsed systematically through new experiments to accurately place them into a quantitative theory. This is a large burden and will take considerable effort and likely rely heavily on the use of mathematical modeling and quantitative experiments for testing. It is, however, a point of conjecture that all of the literature findings that appear to strongly support the subset paradigm can be reinterpreted within qCST.

## 19 BOLD DREAMS FOR A BIOLOGICAL FUTURE?

Mathematical models of natural systems are limited by the accuracy of their parent theory. A good theory offers the equivalent of axioms that describe the system and the rules that when applied govern the predicted consequences. I have adopted that view in trying to frame our immune problem into two components, the cell mechanics (the axioms) and the cellular calculus (the rules and operations for deducing the consequences). The interesting challenge presented by immunology and biology, in general, is that these models have to deal with so many levels of scale and we have to capture these operators into mathematical expression that ideally translates results between levels.

When developing and describing models for these processes, I find the conventional calculus unintuitive as it does not match my experience of biology and frequently creates solutions that offer little insight for the job at hand. For example, an ordinary differential equation (ODE) can fit perfectly to exponential cell growth systems but does so based on incorrect assumptions.^55^ It will predict, wrongly, that a proportion of cells are capable of infinitely short division times. An ODE will also naturally assume that the genesis of all variation is exponential and memoryless, and these two assumptions are almost never true in biology.^55^ Of course, these examples can, and have been, corrected in more complex models, to more closely match the biology, but this requires further ad hoc additions to recapture the specific behavior and still may not lead to predictions far beyond what is already known. The calculus in use today was invented almost 400 years ago with operators well suited to independent forces and uniform particles that are physically unchanged by interaction. Is it possible to imagine a new approach and invent a biological calculus that is intuitive and powerful and reflects correct component operation at all levels? That is, could we, instead of forcing biology to conform to mathematical operations suited to physics, invent an intuitive mathematical framework for biology built on established rules of cellular function that naturally operates between multiple levels of scale?

As encouragement for a general scaling calculus, it is notable that there are patterns in biology, such as creation, randomization, selection, and evolution that repeat at multiple levels. It is easy to see how a variant of the cellular calculus could be adapted to species evolution or human group dynamics. Every person is constructed by identical processes but is a unique combination of organismal level component machines (ie, brain, muscles, heart) that see them behave differently under identical situations. While we might not be able to predict the behavior of an individual, we might do a reasonable job of predicting the behavior of populations and the diverse consequences that arise over time. What if we look to lower levels? A related strategy might successfully describe molecular construction, molecular interactions, and reaction products over time as an evolutionary process. It is tempting to keep going down in levels to the atomic and subatomic. Could we revisit the axiom that atoms are all identical and posit instead they might be the product of slight variations in construction that sees them heterogeneous at minute scale and conforming to some elementary version of the biologically inspired calculus? There might be interesting consequences if so.

Irrespective of how many levels might be connected, this dream of capturing a universal multiscale evolutionary calculus carried to its logical endpoint raises the possibility of bespoke logical circuits and analog computer designs that use probabilistic rather than binary switching and would be capable of powerful deductive possibilities for immunology and many other biological, and possibly physical, applications.

## 20 CONCLUSION

At the time Burnet was formulating CST, the most popular paradigm for solving how antibodies could be specific for such a broad range of antigens was that it must be a property of the antibody molecule itself. The dominant theory being antibody could adapt itself, by folding, to any foreign shape.^91^ Burnet's achievement was to raise investigation of immune specificity from the molecular paradigm, which we now know, could not lead to an answer, to that of a cell population‐dependent response. Burnet also proposed the use of random combinations to solve the coding problem for immense diversity. It is satisfying, and seems in the spirit of the original theory, that the modifications discussed here preserve the reliance of CST on a population of responding cells to complete the immune response and that random combinations are further exploited to regulate the control and generation of multiple effector fates.

## CONFLICT OF INTEREST

I have no conflict of interest for this paper.
